# Mesoscopic 3D imaging of pancreatic cancer and Langerhans islets based on tissue autofluorescence

**DOI:** 10.1038/s41598-020-74616-6

**Published:** 2020-10-26

**Authors:** Max Hahn, Christoffer Nord, Oskar Franklin, Tomas Alanentalo, Martin Isaksson Mettävainio, Federico Morini, Maria Eriksson, Olle Korsgren, Malin Sund, Ulf Ahlgren

**Affiliations:** 1 Umeå Centre for Molecular Medicine, Umeå University, Johan Bures väg 12, 901 87 Umeå, Sweden; 2 Department of Medical Biosciences, Umeå University, Umeå, Sweden; 3 Department of Immunology, Genetics and Pathology, The Rudbeck Laboratory, Uppsala University, Uppsala, Sweden; 4 Department of Surgical and Perioperative Sciences/Surgery, Umeå University Hospital, Umeå, Sweden

**Keywords:** Pancreatic cancer, Optical imaging, Pancreas, Pancreatic cancer, Islets of Langerhans

## Abstract

The possibility to assess pancreatic anatomy with microscopic resolution in three dimensions (3D) would significantly add to pathological analyses of disease processes. Pancreatic ductal adenocarcinoma (PDAC) has a bleak prognosis with over 90% of the patients dying within 5 years after diagnosis. Cure can be achieved by surgical resection, but the efficiency remains drearily low. Here we demonstrate a method that without prior immunohistochemical labelling provides insight into the 3D microenvironment and spread of PDAC and premalignant cysts in intact surgical biopsies. The method is based solely on the autofluorescent properties of the investigated tissues using optical projection tomography and/or light-sheet fluorescence microscopy. It does not interfere with subsequent histopathological analysis and may facilitate identification of tumor-free resection margins within hours. We further demonstrate how the developed approach can be used to assess individual volumes and numbers of the islets of Langerhans in unprecedently large biopsies of human pancreatic tissue, thus providing a new means by which remaining islet mass may be assessed in settings of diabetes. Generally, the method may provide a fast approach to provide new anatomical insight into pancreatic pathophysiology.

## Introduction

Modern optical imaging technologies are starting to show potential as tools for obtaining 3D insight into pancreatic disease based on whole mount immunolabelling of pancreatic tissues^1,2^. Still, there are several limitations for their use in clinical practice. Firstly, lengthy protocols are required to ensure reagent/antibody penetration into larger pancreatic tissue biopsies, with an upper limit in the lower mm rather than the cm range. Secondly, employing whole mount immunohistochemical protocols may hamper or prevent subsequent routine histopathological analyses of tissue sections.

Although 3D imaging techniques, such as optical projection tomography (OPT)^3^ that is based on the attenuation contrast of light^4^ have been used for 3D imaging of mouse embryos without labels, few studies, if any, have used the autofluorescent (AF) properties of the pancreas for 3D assessments of mesoscopic sized (mm-cm scale) tissue specimens. OPT could be described as a CT scanner allowing for 3D imaging of biological specimen that employs light in the visible or near infrared part of the spectrum instead of using x-rays. Similarly, light sheet fluorescent microscopy (LSFM), aka selective plane illumination microscopy (SPIM)^5–7^ has emerged as a powerful tool for high-resolution studies of optically cleared specimen (for a comprehensive review of OPT versus LSFM see Liu et al.^8^). In contrast to OPT, LSFM acquires data by performing illumination and detection of the sample in two distinct optical paths that are organized orthogonally to each other. Hereby, a stack of optical sections is produced by either moving a laser generated light sheet through the specimen, alternatively by moving the specimen through the light sheet. Although benefitting from higher resolution scans, the latter platform has limitations in terms of maximum tissue-size and non-isotropic voxels for quantitative assessments. Scanning times are significantly longer for LSFM compared to OPT for similar sized specimen, depending on the applied resolution. Generalized, OPT may be more appropriate for larger samples at lower magnification, relating to scan times and data volumes, and subsequently for larger numbers of samples. LSFM in turn may be more appropriate for higher resolution studies (of smaller numbers of samples). Still, the techniques are to a high degree interchangeable and can often substitute each other^9^. Previously, both techniques have been successfully implemented in studies of pancreatic islets in various models of rodent diabetes models based on specific antibody labelling^10–14^.

Autofluorescent (AF) properties of tissues have been used for decades as a source of contrast to demarcate specific cells, cellular constituents or cellular processes. As such, AF has been used to study various pathological features on tissue sections. The source of pancreatic AF is multifaceted and may originate from different compounds or molecules such as NADPH, laminins, collagens, elastin, porfyrin and lipofuscines (for review see e.g.^15^). An important feature of naturally occurring AF is that different compounds have emission profiles that may enable channel separation of tissues and/or proteins. For example, collagens and elastins contribute to blood vessel structures and are strong sources of AF^16^. Lipofuscin is a compound pigment formed as the result of lysosomal digestion that contain lipids, sugars and proteins. It has strong AF properties and is found in a variety of tissues including pancreatic islets. AF from lipofuscin bodies has moreover been used to study the longevity of islets in pancreatic tissue from diseased donors^17^. Detection of specific AF properties has also been suggested as a means for cancer screening of various tissues^18^, but not for assessing tumour spread in intact surgical biopsies of human pancreas.

Over the past decades, no improvement in survival has been achieved for PDAC and the incidence of this cancer form is increasing^19^. Curative treatment can only be achieved through radical surgery combined with adjuvant chemotherapy^20^. A key issue in this regard is obtaining tumour-free resection margins, which are normally determined by histopathological analyses on resected tissue sections. PDACs grow within a dense tumour stroma and cancer cells are spread at relatively large distances within this matrix. The extent of the tumour is therefore often underestimated leading to false negative resection margins^21^. Using OPT and LSFM imaging, we here present protocols that facilitate assessments of tumor characteristics and extent without adversely affecting routine histopathological analyses, which instead could be guided by these protocols. Using endogenous tissue AF to obtain tissue contrast, the whole protocol including image display and analysis can be performed within hours. As such it holds potential to become a valuable addition to today´s toolbox of histopathological techniques for identification and characterization of pancreatic cancers and for determination of islet mass distributions.

## Materials and methods

The study was conducted in accordance with the Helsinki declaration of 1975 and approved by the ethical committee of Northern Sweden (2016/384-31, 2019-04593 and 09-175M/2009-1378-31). All patients signed informed consent for tissues to be collected. Pancreatic cancer tissue was collected during pancreatic cancer surgery from patients admitted to the Department of Surgery at Umeå University Hospital, Sweden or from diseased donors within the framework for the Nordic Network for Clinical Islet Transplantation (NNCIT).

### Samples

Biopsies from the displayed tumour specimens were collected at surgery and fixed in formalin. The biopsy displayed in Fig. 2 was collected from a patient with histopathologically verified PDAC and the biopsy displayed in Fig. 3 was collected from a patient with intraductal papillary mucinous neoplasia (IPMN) without evidence of invasive cancer. Tissue biopsies for assessments of islet mass distribution were isolated from formalin fixed pancreata post-mortem (Fig. 5, Supplementary Dataset 1).

### Sample preparation for OPT or LSFM imaging

**Figure 1:**
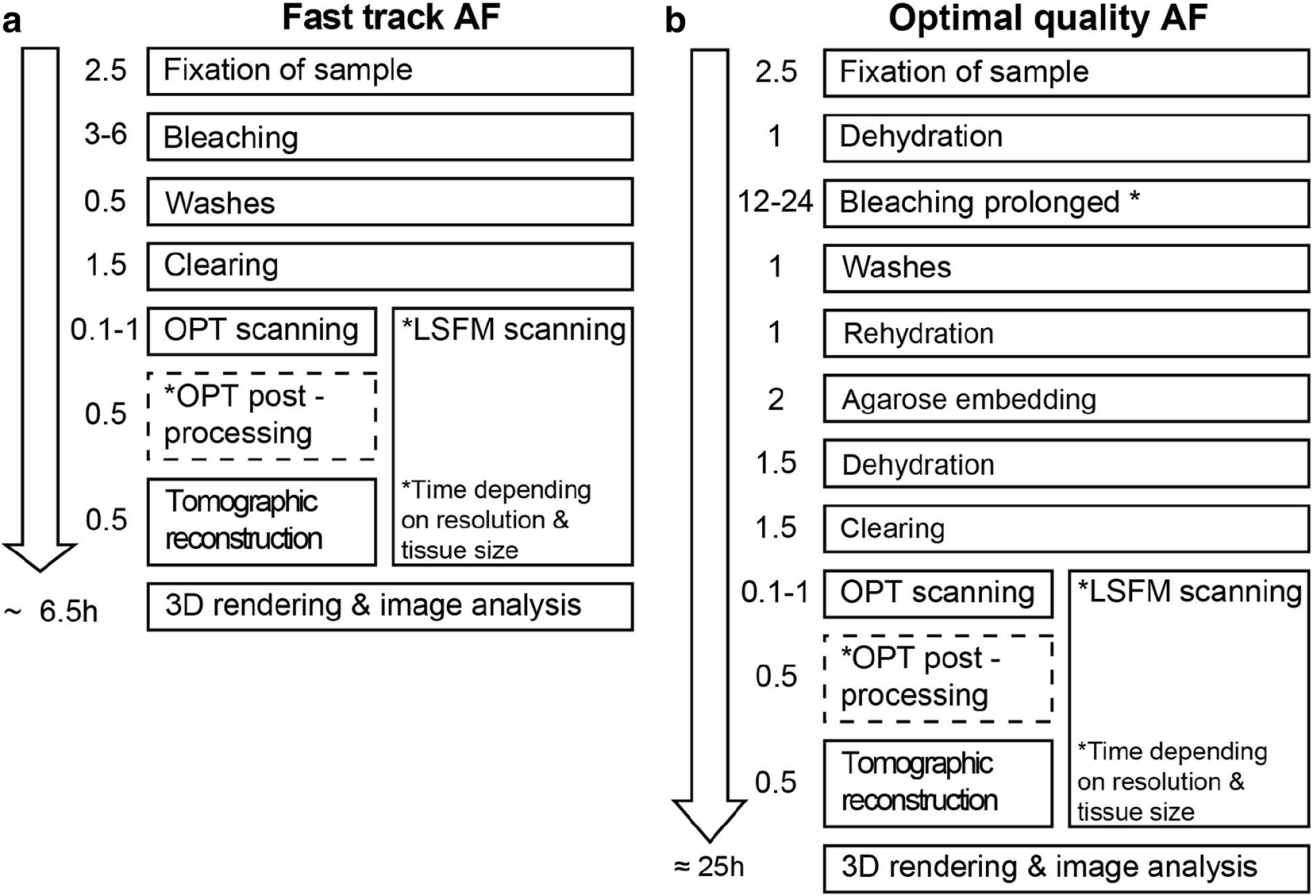
Protocols for label free, AF based imaging of pancreatic tissue biopsies. Schematic outlining two alternative protocols for AF based imaging of pancreatic tissue biopsies (see methods for details). The fast track protocol (**a**) enables 3D analyses of AF features, including generation of full 3D and tomographic data, within in less than 7 h from that the sample is received. In the optimal quality track (**b**) we attempted to optimize every parameter of the protocol for imaging scenarios for which time is not a critical factor.

Approximate time schedules for the described “fast” and “optimal quality” imaging protocols, are displayed in Fig. 1. We recommend using the fast track protocol for smaller (approx. 1 × 1 × 0.5 cm) samples, and samples that require rapid screening. For larger specimen and samples that bear a lot of membranes or adipose tissue, we recommend using the optimal quality protocol to ensure adequate contrast and transparency for post-imaging analysis.

#### FAST TRACK AF protocol

Specimen are fixed in 4% w/vol Paraformaldehyde (PFA, Sigma Aldrich 30525–89-4) at 4 degrees Celsius over a period of 2 h and 30 min. They are then transferred to 5 mL 30% Hydrogen Peroxide vol/vol H2O2 (Merck 7722-84-1) for 30 min at room temperature on rotation in the dark. Samples are afterwards dehydrated in 15 mL Methanol (MeOH, Fisher Chemical 67-56-1) for 30 min, changing solvent every 10 min, and quickly cleared in BABB (Benzyl Alcohol: Benzyl Benzoate = 1:2, CAS 100-51-6 Merck, 120-51-4 Acros Organics respectively) for 1 h and 30 min. During this period the solvent is changed every 30 min. Throughout these two steps samples are rotating at room temperature.

#### OPTIMAL QUALITY AF protocol

Specimen are fixed in 4% w/vol PFA at 4 degrees Celsius over a period of 2 h and 30 min. They are then dehydrated stepwise in Methanol/PBS1X 33-66-100%, with each step lasting 20 min at RT. Samples are then bleached in 10 ml fresh bleaching solution (H2O2 : MeOH : DMSO (Dimethyl sulfoxide 67-68-5, Merck) = 3:2:1) at room temperature in the dark, for 24 h, changing the bleaching solution to a fresh one overnight. Samples are then rehydrated in TBST/MeOH 33-66-100% at room temperature. During dehydration and dehydration samples are kept on rotation. Samples are then mounted in 1.5% w/vol Low melting point SeaPlaque Agarose (39346-81-1 Lonza) as previously described^12^, dehydrated in MeOH for 30 min and cleared in BABB as described above. Samples are subsequently scanned by OPT or LSFM.

### Pancreatic AF based optical projection tomography (OPT) imaging

OPT scanning was performed using an in house built Near Infrared-OPT setup as described^12^. The samples were imaged in different wavelengths using filters of Ex: 425/60 nm & Em: 480 nm LP, Ex: 480/40 nm & Em: 510 nm LP, Ex: HQ 565/30 nm & Em: HQ 620/60 nm, Ex: 628/40 nm & Em: 692/40 nm, Ex: 640/20 nm & Em: 680/30 nm and Ex: HQ 665/45 nm & Em: HQ 725/50 nm.

### Pancreatic AF based light sheet fluorescence microscopy (LSFM) imaging

**Figure 2:**
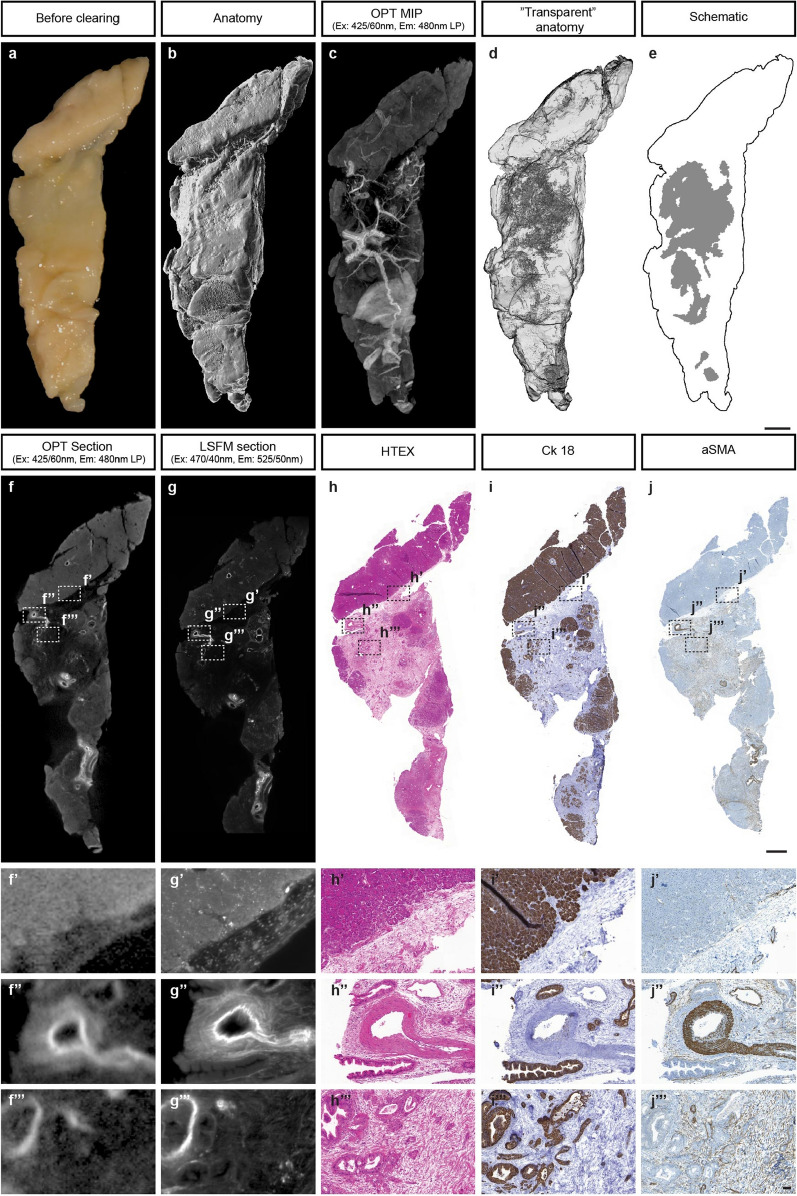
AF-based 3D analyses of PDAC biopsies facilitates assessments of tumor delineation and vascularisation. (**a**–**j**) Biopsy collected from a patient with PDAC verified by histopathology. (**a**) Bright field image of uncleared biopsy after fixation. (**b**) Segmented anatomy (OPT-generated iso-surface reconstruction) of the biopsy based on its endogenous fluorescence. (**c**) Maximum intensity projection (MIP) view of the tomographic data set in a part of the spectrum enabling detection of vascular and ductal structures. Note the pronounced vascularisation into the PDAC tissue. (**d**) “Transparent anatomy” segmentation based on tissue AF. The delineation of the tumor tissue is based on low AF intensity regions and appears like a grey mesh that corresponds to histopathological analyses of the same tissue (**h**–**j**). (**e**) Schematic outline of the visualized tumor volume. (**f**) Tomographic OPT section through the PDAC biopsy seen in (**a**). (**g**) LSFM section corresponding to the section seen in (**f**) of the same specimen. (**h**) HTEX stained paraffin section of the sample seen in (**a**) approximately corresponding to the tomographic sections in (**f**,**g**). Note, the delineation of the tumor tissue corresponds, section by section, with the outline of the tumor as visualized in (**d**). (**i**) Tissue section consecutive to (**h**), stained for Ck18 as marker for epithelial tissue. (**j**) Section consecutive to (**h**,**i**), stained for aSMA as a marker for major blood vessels. Abbreviations; OPT, optical projection tomography: LSFM, light sheet fluorescence microscopy: HTEX, hematoxylin/eosin; Ck18, cytokeratin 18: aSMA, smooth muscle alpha-actin. Scale bar in (**e**) is 1500 μm in (**a**–**e**), scale bar in (**j**) is 1000 μm in (**f**–**j**) and scale bar in (**j′″**) is 50 μm in (**f′**–**j′″**).

Specimen that were OPT processed and imaged (see above), were reimaged by a LaVision biotech 2nd generation UltraMicroscope II (LaVision BioTec GmbH, Germany). The samples mounted in agarose were either trimmed in BABB (see sample preparation) to fit into the UltraMicroscope II sample holder or glued onto the sample holder before image acquisition. Samples displayed in Fig. 2 and Fig. S2 were imaged using filter sets of Ex: 470/40 nm & Em: 525/50 nm and Ex: 650/45 nm & Em: 750/60 nm but were tested in a range of additional wavelengths producing results similar to Fig. S1. Samples (detailed information in Supplementary Dataset 1) were imaged with a 1 × Olympus objective (Olympus PLAPO 2XC) coupled to a Olympus MVX10 zoom body, providing 0.63 × up to 6.3 × with a lens correctd dipping cap MVPLAPO 2 × DC DBE objective (LaVision Biotec BioTec GmbH, Germany). Tile scans with 20% overlap along the longitudinal y axis were obtained. In general, exposure time was 582–843 ms with a light sheet width of 40–50%, 3.87 µm thickness and 0.14 NA using a z-step of 4–5 µm at 0.80 X magnification.

### Post-image processing and Image analysis

**Figure 3:**
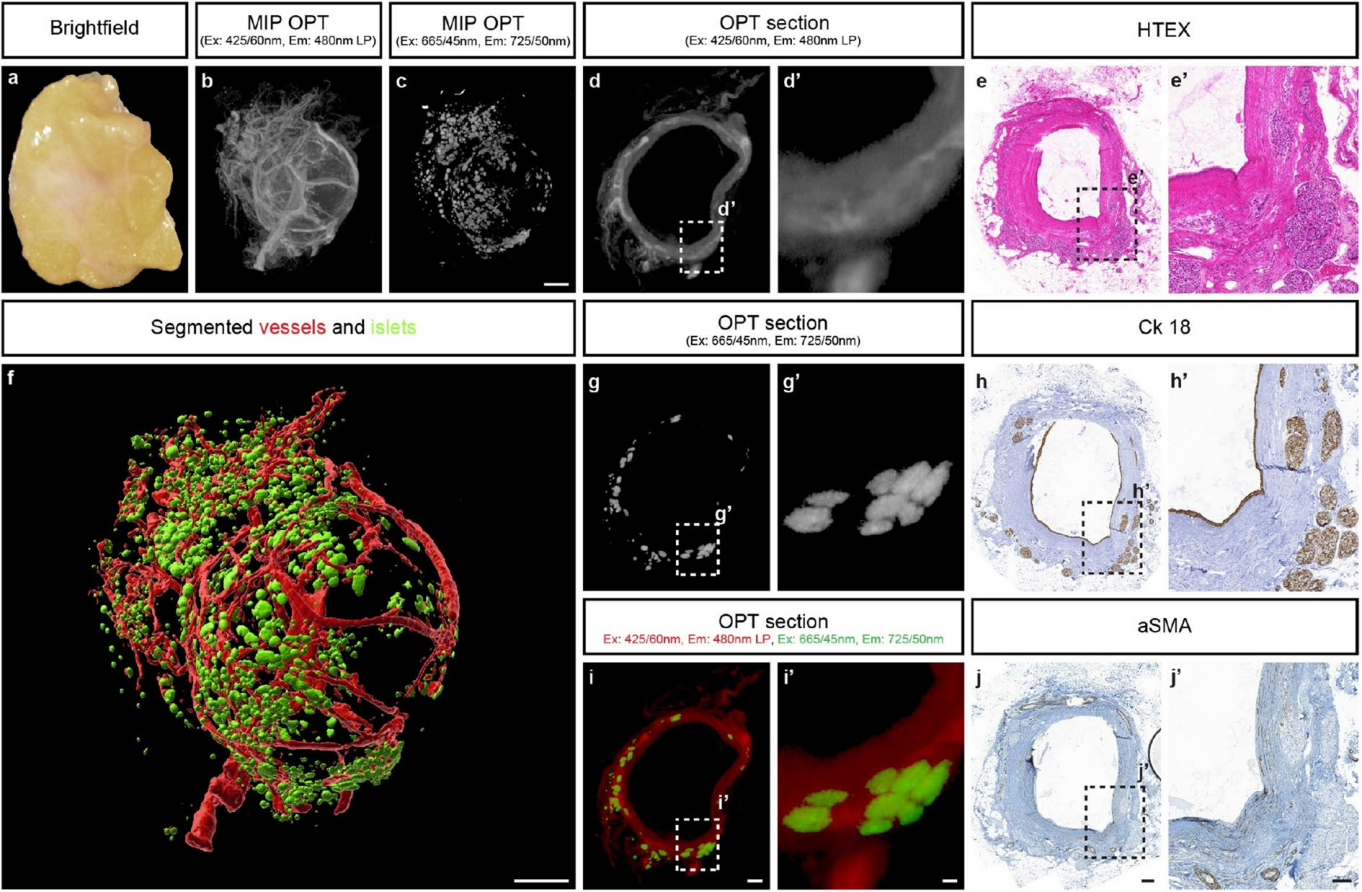
3D imaging of IPMN cyst with associated vessels and islets based on its AF properties. (**a**–**h**) A pancreatic cyst from a patient with intraductal papillary mucinous neoplasia. (**a**) Bright field image of the cyst after fixation. (**b**) Maximum intensity projection (MIP) view of the tomographic data set in a part of the spectrum enabling detection of vascular and structures. (**c**) MIP view of the cyst (in **a**) in the Near infrared (NIR) spectrum illustrating the possibility to visualize the islets of Langerhans based on their AF properties (see also Fig. S1). (**d**) Tomographic section through the cyst approximately corresponding to the section in (**e**) showing the cyst wall and associated vessels. (**e**) HTEX staining through a cross section of the cyst post OPT-imaging. (**f**) Iso-surface reconstruction of the cyst showing vasculature (red) and islets of Langerhans (green), see also Video S6. (**g**) Tomographic section through the cyst corresponding to (**d**) showing the islets of Langerhans. (**i**) Overlay of the tomographic sections in (**d**, red) and (**g**, green). (**h**) Tissue section consecutive to (**e**), stained for Ck18 as marker for epithelial tissue. (**j**) Section consecutive to (**e**), stained for aSMA as a marker for major blood vessels. (**d′**,**e′**,**g′**–**i′**), blow up views corresponding to the insets in (**d**,**e**,**g**–**j**). Abbreviations; Ex, Excitation: Em, Emission: HTEX, hematoxylin/eosin: Ck18, Cytokeratin 18: aSMA, smooth muscle alpha-actin. Scale bar in (**c**) is 1000 μm in (**a**–**c**), scale bar in (**i**) is 500 μm in (**d**,**g**,**i**), scale bar in (**i′**) is 100 μm in (**d′**,**g′**,**i′**), scale bar in (**j**) is 200 μm in (**e**,**h**,**j**), scale bar in (**j′**) is 100 μm in (**e′**,**h′**,**j′**). Scale bar in (**f**) is 1000 μm.

For post-OPT processing (detailed information see Supplementary Dataset 1), ranges of pixel values of subsequent OPT image data were cut to increase signal-to-background ratio, a contrast limited adaptive histogram equalization (CLAHE)^22^ algorithm with a tile size of 32 × 32 and a post-acquisition misalignment detection and correction using Discrete Fourier Transform Alignment (DFTA)^23^ was applied. The processed frontal projections were then reconstructed to tomographic sections using the NRecon v1.6.9.18 software (Skyscan, Belgium). Tomographic OPT sections and resultant LSFM image data was converted, visualized and analyzed using Imaris File Converter and Imaris 9.5. (Bitplane, UK). Using the automatic surface algorithm in Imaris for the lowest wavelength of autofluorescence the anatomy was segmented (Fig. 2b), and the displayed texture was adjusted to make the segmented anatomy “transparent” (Fig. 2d). The same automatic surface algorithm with different creation parameters was applied to segment out vessel/duct like tubular structures (Fig. 2f). For the signal intensity spot analysis (Video S2) the Imaris automatic spot algorithm was applied to the maximum intensity projection (MIP) data and statistically color coded based on the sum of intensities for the investigated channel, OPT; Ex: 425/60 nm, Em: 480 nm LP and for LSFM; Ex: 470/40 nm, Em: 525/50 nm. For OPT-base islet visualization and quantification (Figs. 3f and 5 and 6), the near infrared imaged OPT samples were baseline subtracted and automatically 3D surfaced with a voxel number filter. Artefacts and tiny hairs were removed for quantification and overall statistics were exported to excel file format. The average 3D islet diameter (average length of x, y and z per islet) was used as a categorization norm to compare with data from published stereological islet assessments^24^.

### Immunohistochemical stainings

The formalin-fixed biopsies were paraffin-embedded and sectioned for Haematoxylin & Eosin (HTEX) and immunohistochemical staining. Primary antibodies used were; rabbit anti-cytokeratin 18 (CK18) at 1/50 dilution (ab52948, Abcam, Cambridge, United Kingdom) and rabbit anti-alpha smooth muscle actin (aSMA) at 1/200 dilution (ab5694, Abcam, Cambridge, United Kingdom). Antibody staining’s were performed using an automated Ventana Benchmark staining machine (Ventana Medical Systems, Tucson, AZ, USA). Antigens were retrieved with citrate buffer at pH 6.0 for 8 min at 94 °C. For validation of islet AF on cryosections, guinea pig anti-Insulin (A0564, DAKO) at 1/500 and Alexa Fluor 488 goat anti-guinea pig, dilution 1/500 (A11073, Invitrogen Thermo Fisher Scientific) were used.

## Results

### A protocol for AF based optical 3D assessments of pancreatic tissue

**Figure 4:**
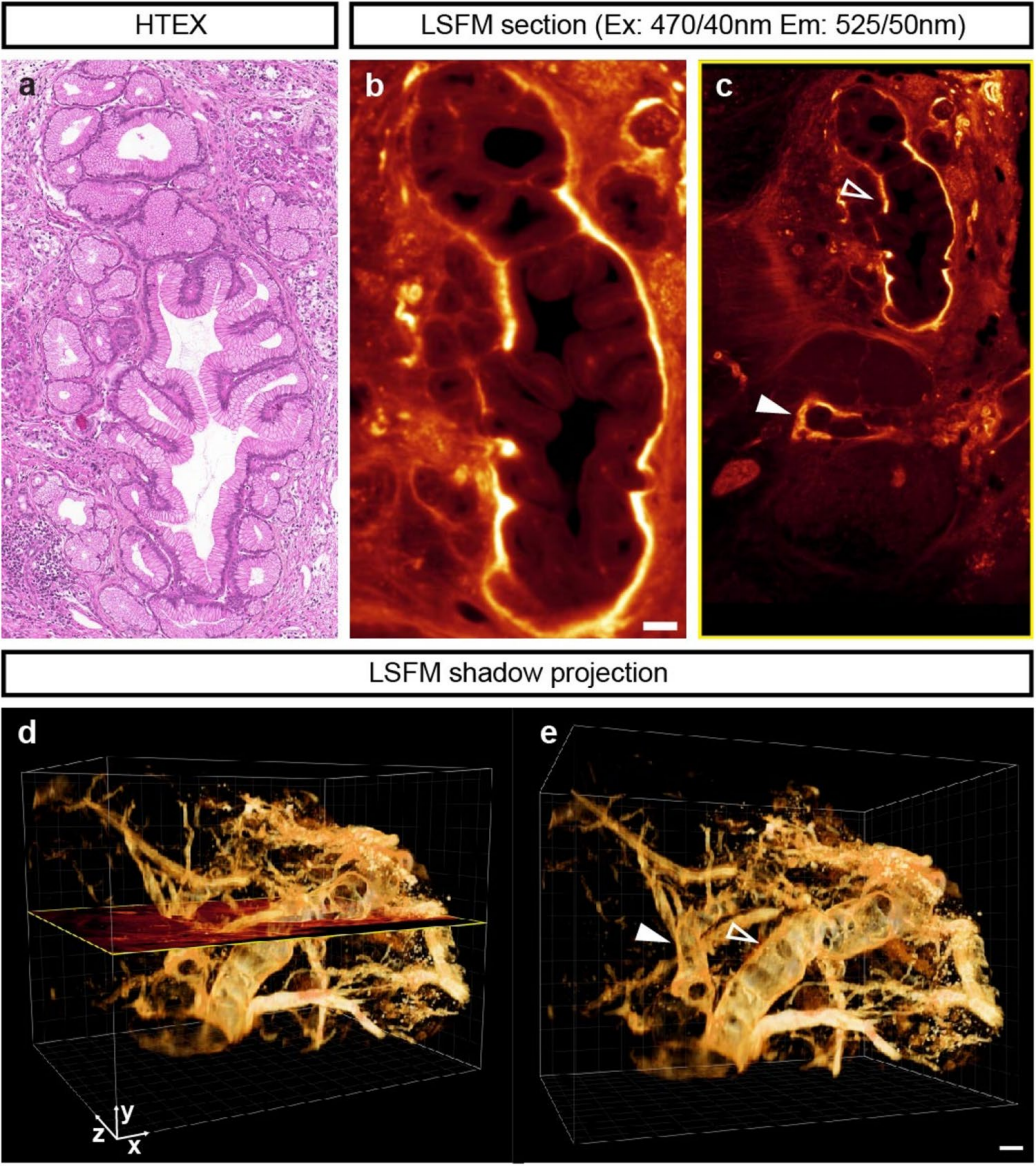
Histopathological assessments of PanIN tissue regions can be coupled to mesoscopic 3D assessments based on AF. (**a**) HTEX staining of a low grade PanIN region from the specimen displayed in Fig. 1. (**b**) LSFM Sect. (3.8 μm) of the same uncut specimen corresponding to the HTEX section in (**a**). (**c**) Larger area encompassing the structure seen in (**b**, open arrow). (**d**,**e**) Shadow projections based on the AF signal seen in (**b**,**c**), showing the 3D structure of the PanIN tissue seen in (**a**). The section plane in (**d**) corresponds to (**c**) and the open and closed arrow in (**e**) corresponds to those in (**c**). Abbreviations; Ex, Excitation: Em, Emission: HTEX, hematoxylin/eosin. Scale bar in (**e**) is 50 μm in (**a**,**c**–**e**), scale bar in (**b**) is 20 μm.

**Figure 5:**
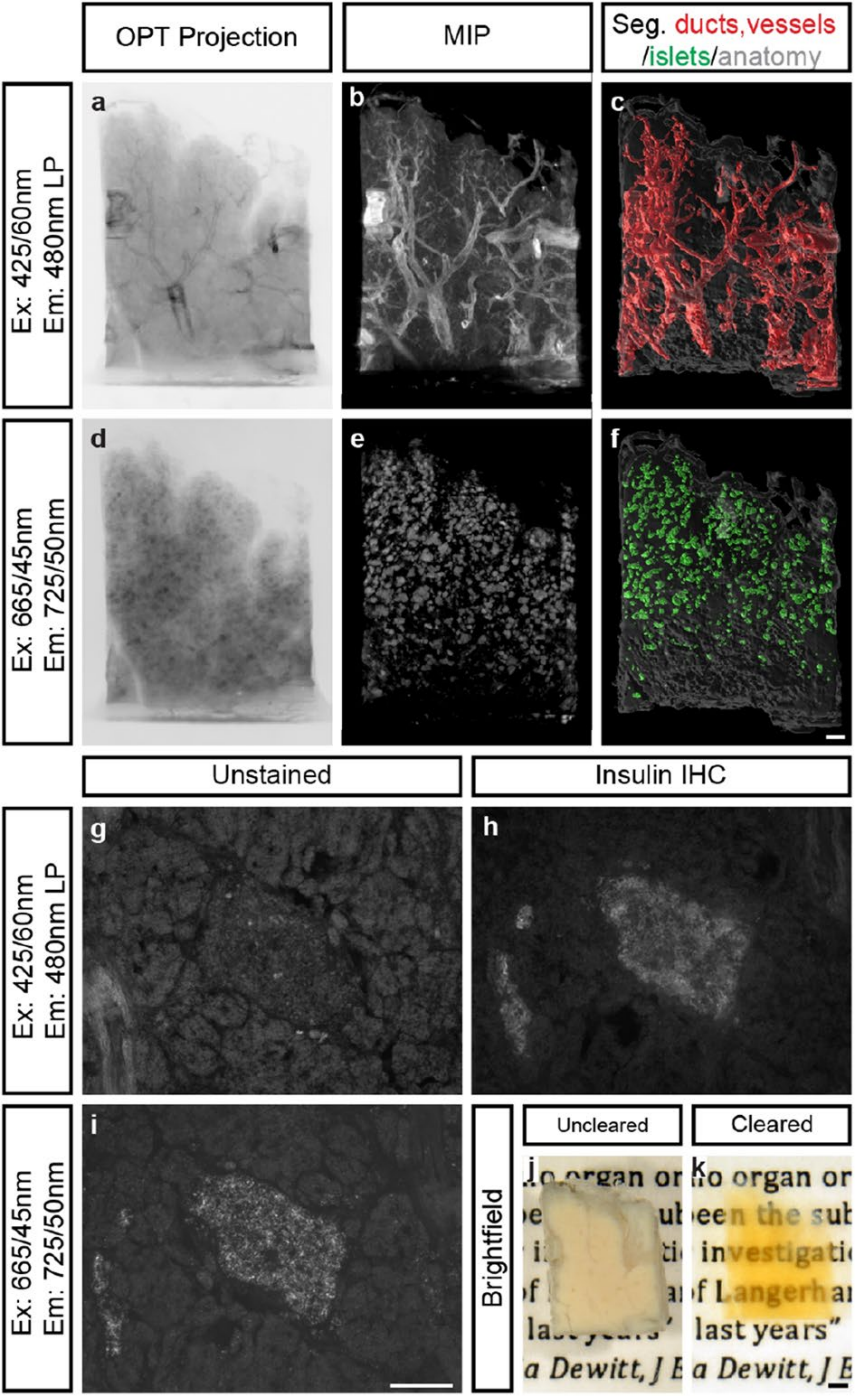
Imaging of islet mass distribution based on endogenous islet AF. (**a**–**f**) Images displaying a tissue biopsy from a non-diabetic donor based on their AF properties using indicated filter sets. (**a**–**c**) Raw OPT projection view (**a**), post-processed maximum projection intensity (MIP) image (**b**) and segmentation of the AF signal delineating tubular structures (**c**, red). (**d**–**e**) Raw OPT projection view (**d**), post-processed maximum projection intensity (MIP) image (**e**) and segmentation of the AF signal delineating the Islets of Langerhans (**f**, green). (**g**–**i**) Epifluorescence images of cryosections (20 μm) showing the islet specificity of the AF signal using the indicated filter sets. (**g**) Unstained control section showing absence of AF in the indicated spectrum. (**h**) Consecutive section stained for insulin. (**i**) Unstained consecutive section to (**g**,**h**) visualized in the indicated spectrum showing AF specific to the islet, compare i and h. Scale bar in (**f**) is 500 μm in (**a**–**f**), scale bar in (**i**) is 200 μm in (**g**–**i**) and scale bar in (**k**) is 1000 μm in (**j**,**k**).

By scanning optically cleared pancreatic specimen in emission spectra ranging from 425 to 680 nm, we initially assessed which parts of the spectrum provide channel separation between recognisable features in PDAC and normal pancreatic tissue (Fig. S1). When implementing either a “fast track” protocol that could be used in conjunction with clinical investigations, or a protocol in which every parameter of the tissue and image acquisition process was optimized (see “Materials and methods” section and Fig. 1), 3D imaging and analysis of > cm^3^-sized tissue biopsies was performed (See Figs. 2, 3, 4, 5).

### AF based assessments of tumour microenvironment

As exemplified in Fig. 2, a PDAC tissue sample revealed noticeable tubular features as well as areas of higher and lower intensity (Fig. 2a-e), when subjected to tissue clearing and OPT and LSFM imaging (see also Fig. S2). Dual modality imaging of the same specimen with OPT and LSFM revealed similar features (Fig. 2f,g), although LSFM produces higher resolution images albeit at the expense of significantly longer scanning times and non iso-tropic voxels (see also Fig. S2 and Videos S1, and S2). At the implemented wavelengths vascular structures appeared as prominent features in the tomographic data sets, allowing individual vessels and their trajectories to be chartered in interactive 3D data sets of the biopsies (Fig. 2b and Video S3). PDACs are generally characterised as scarcely vascularized, a feature believed to cause high interstitial tissue pressure and poor systemic treatments effects^25^. In contrast to this, our sample was surprisingly well vascularised on 3D examination.

The normal pancreatic parenchyma displays a considerably higher AF signal intensity than the PDAC tissue. When applying a transparency filter on the iso-surfaced dataset in the Imaris software (see online methods and Supplementary Dataset 1), this low intensity AF region could be visualized in 3D within the entire volume of the biopsy (Fig. 2c and Video S4). The PDAC area is characterized by abundant stroma and extracellular matrix structures seen as a grey mesh in the transparent anatomy (Fig. 2d)^21,26^ (compare Fig. 2d,f). The approximate anatomical outline of the tissue could further be visualized in 3D signal intensity hot-spot histograms, based on the tissue AF signal intensity (Video S4). The outline based on AF closely resembled the outline of the cancer regions as determined by HTEX and Ck18 staining at each level (compare Fig. 2f,g,h,i), whereas the endogenous tissue fluorescence from vascular/tubular structures partly overlapped with aSMA (compare Fig. 2f,g,j). Based on the 2D histopathological information (e.g. HTEX), it is possible to localize the corresponding PDAC regions in the LSFM or OPT data sets. PDAC development is preceded by premalignant lesions that include pancreatic intraepithelial neoplasia (PanINs) and intraductal papillary mucinous neoplasms (IPMNs)^27^. By segmentation of the AF signal, PanIN regions within the tissue appears to exhibit 3D tubular structures with connecting branches (Fig. 4 and Video S5).

**Figure 6:**
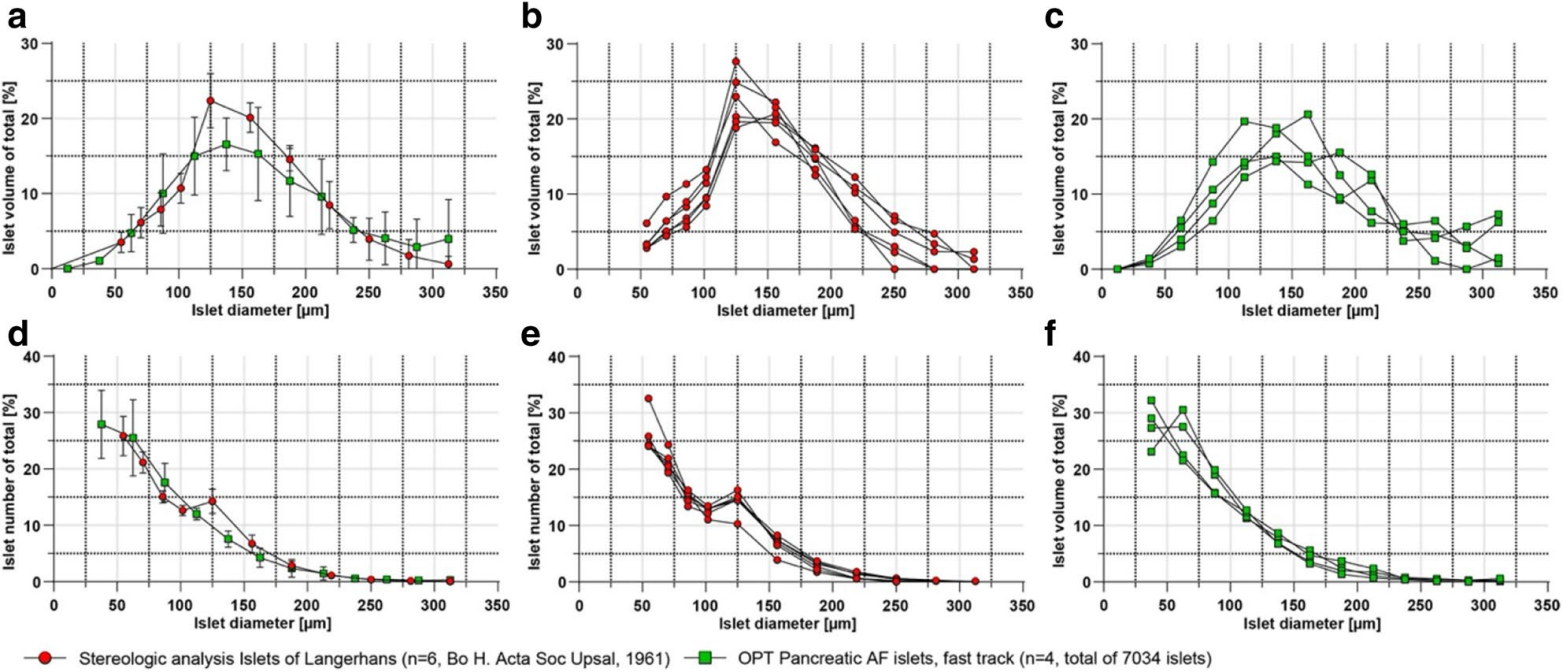
OPT data of islet volume and number distributions correlate with stereological assessments. (**a**) Graphs displaying islet mass distribution from OPT measurements (displayed as islet diameters calculated from average length of the individual islets x, y and z axis, green) into size categories of a total of 7034 islets (n = 4 biopsies), compared to stereological assessments as described by Hellman et al., (red). (**b**) The same stereological data as in (**a**) but from individual pancreata. (**c**) The same OPT-data as in (**a**) but from individual biopsies. (**d**) The same data as in (**a**) but displayed as the number of islets falling within each size category. (**e**) The same stereological data as in (**d**) but from individual pancreata. (**f**) The same OPT-data as in (**a**) but from individual biopsies. Notwithstanding that the OPT material was collected from different regions of the pancreata (see methods), it is well in line with previous stereological assessments. The data in (**a**,**d**) are presented as mean ± SEM.

When applying the proposed imaging schemes to an IPMN cyst (Fig. 3), vessel trajectories could be followed similarly to the PDAC specimen (Fig. 3b,f and Video S6). These surrounded the spherical cyst, which in itself did not produce an AF signal. Interestingly, when detecting AF in the near infrared (NIR) spectrum (Ex: 665/45 nm Em: 725/50 nm for OPT and Ex: 650/45 nm Em: 750/60 nm for LSFM), islets of Langerhans could easily be segmented (Fig. 3c,f, see also Figs. 5 and 6). In HTEX staining of the sectioned lesion, the vascular and endocrine components could be directly matched with tomographic slices (Fig. 3d,e,g,i). Whereas the core of the cyst did not display an AF signal in the investigated spectra, immunohistochemistry revealed a hollow structure with an epithelial lining and a relatively thick cyst wall, filled with mucin (Fig. 3h,j). Similar results were obtained by LSFM analyses (Video S7).

### AF based visualisation and quantification of the islets of Langerhans

Quantification of islets in larger human pancreatic tissue volumes is challenging and most commonly involves extrapolation of 2D data resulting from labour-consuming stereological assessments^28^. As noted, when analysing the surgical specimens, detection and segmentation of islets within pancreatic tissue is feasible based on their AF signal, which was confirmed by analyses of normal pancreas tissue obtained from deceased donors (Fig. 5). Hence, by applying a NIR filter set in the OPT scanner, the endogenous fluorescence from the islets was clearly visualized (see Fig. 5a–c,d–f, Fig. S1 and Video S8). Similar results were obtained for LSFM imaging (Fig. S2). The AF signal was determined to be islet specific in this part of the spectrum, by comparing it to insulin antibody stained tissue sections (Fig. 5g–i). Implementing our previously developed OPT post-processing routines (see Material and Methods) islet volumes could be segmented, and the islet numbers and their individual volumes calculated. Notwithstanding the distinct origin of OPT-analysed material (see Supplementary Dataset 1) and the relatively low AF signal intensity, the obtained number of islets and their distribution characteristics are well in line with comprehensive stereological assessments (Fig. 6)^24^. Taken together, endogenous islet AF may be used to assess islet mass distribution in unprecedently large tissue biopsies of the human pancreas.

## Discussion

Although mesoscopic imaging approaches has been demonstrated as useful modalities for pathological assessments of human tissues, these have largely relied on staining’s of the investigated samples. For example, Glaser et al., demonstrated how an optimized light sheet microscope could be used for non-destructive pathological assessments of (non-pancreatic) clinical specimens stained by fluorescence emitting dye^29^, whereas Nojima et al., demonstrated the utility of LSFM in histopathological analyses of a wide range of antibody stained human tissues^30^. In this short report, we demonstrate the utility of mesoscopic imaging of AF features in optically cleared pancreatic tissue specimens to assess pancreatic anatomy/pathology without the need for prior cell/tissue labelling schemes. We propose that the method could become a useful complement to current routine histopathology in a wide range of pancreatic aberrations, since data can be obtained in a short time without adversely affecting tissue morphology for subsequent immunohistochemistry (see Figs. 2h–j and 3e,h,j). Notwithstanding the limited sample number, the demonstrated possibility to visualize the PDAC morphology in 3D solely based on AF properties of the tissue may have significant implications. Most importantly the developed imaging schemes may develop into a clinically useful tool for analyses of specific regions of interest to facilitate delineation of free resection margins after cancer surgery. In addition, an improved general understanding of 3D tumour morphology at the current resolution could be translated to better evaluation of non-invasive imaging approaches. Further, the possibility to study the individual vessel trajectories in relation to tumour volume may be highly useful to increase our understanding of PDAC vascularization and how to overcome problems related to drug delivery in these cancers.

Although it has been described that islets grafted to the anterior chamber of the eye display AF properties up to 600 nm in vivo^31^, the high signal to noise ratio between islet AF and surrounding pancreatic tissue in the red to the NIR part of the spectrum has to the best of our knowledge not been described previously. In our study, we observed droplet like regions of islet AF over a broad range of the spectrum (excitation maxima tested between 480 and 710 nm). A plausible source candidate for this broad fluorescence profile are lipofuscin-like lipopigments, which are present in highly secretory endocrine cells^15^. Notably, these photoinduced fluorescent granules provided sufficient signal to noise ratio for quantification of islet volumes with 3D imaging tools throughout the investigated tissue in the NIR spectrum, which was challenging at lower wavelengths. Large scale analyses of islet mass distributions in material from deceased diabetic donors (be it type 1 or type 2 diabetes) may contribute to a better understanding of the relationship between remaining islet mass and disease development. Despite the striking resemblance with stereological data from previous studies, the possibility to evaluate the accuracy of these analysis remains limited for large tissue volumes until reliable whole mount immunolabelling protocols for specimen on the current scale are developed.

Since the imaging technology required to perform the described analyses is either commercially available (LSFM) or built to a relatively low cost^32–34^ (see also www.mesospim.org), it should be possible to establish the proposed analysis schemes as a complement to routine procedures in most pathology laboratories. Given the dramatic development in tissue clearing procedures during the past decade (for review see Matryba et al.^35^), it is possible that these procedures can be further refined, facilitating studies of larger tissue samples with even shorter processing times and increased quality. Finally, the described protocols could be directly translated to study other organs and tissues, depending on their AF properties. Our preliminary data suggest that structures such as nerves, striated musculature and vessels may be studied also in other tissues without prior labelling schemes implementing the outlined procedures.

In summary, the developed protocols enable a fast method to assess different anatomical structures such as; vessels, ducts and tumour delineation within mesoscopic-sized pancreatic samples without the need for prior labelling schemes and without negatively affecting histology or subsequent immunohistochemical assays.

## Supplementary information

Supplementary Information 1.

Supplementary Figure S1.

Supplementary Figure S2.

Supplementary Video S1.

Supplementary Video S2.

Supplementary Video S3.

Supplementary Video S4.

Supplementary Video S5.

Supplementary Video S6.

Supplementary Video S7.

Supplementary Video S8.

Supplementary Table S1.

## Data Availability

Raw and processed imaging datasets acquired by NIR-OPT and LSFM on all samples displayed are available upon reasonable request. The software used for OPT image processing are available from the authors upon request subject to an MTA.
