# Supplementary material for: Mesoscopic 3D imaging of pancreatic cancer and Langerhans islets based on tissue autofluorescence
Source: Sci Rep. 2020 Oct 26;10:18246. doi: 10.1038/s41598-020-74616-6 (PMC7588461; doi:10.1038/s41598-020-74616-6)
Supplement: Supplementary file 1 — Supplementary Information 1. [file 41598_2020_74616_MOESM1_ESM.pdf]

Mesoscopic 3D imaging of pancreatic cancer and Langerhans islets based on tissue autofluorescence

Max Hahn[1], Christoffer Nord[1], Oskar Franklin[2], Tomas Alanentalo[1], Martin Isaksson Mettävainio[2], Federico Morini[1], Maria Eriksson[1], Olle Korsgren[3], Malin Sund[2, 4, *] & Ulf Ahlgren[1, *].

[1]Umeå Centre for Molecular Medicine, Umeå University, Sweden
[2]Dept. of Medical Biosciences, Umeå University, Sweden
[3]Dept. of Immunology, Genetics and Pathology, The Rudbeck Laboratory, Uppsala University, Sweden.
[4]Dept. of Surgical and Perioperative Sciences/Surgery, Umeå University Sweden.

Supplementary figures

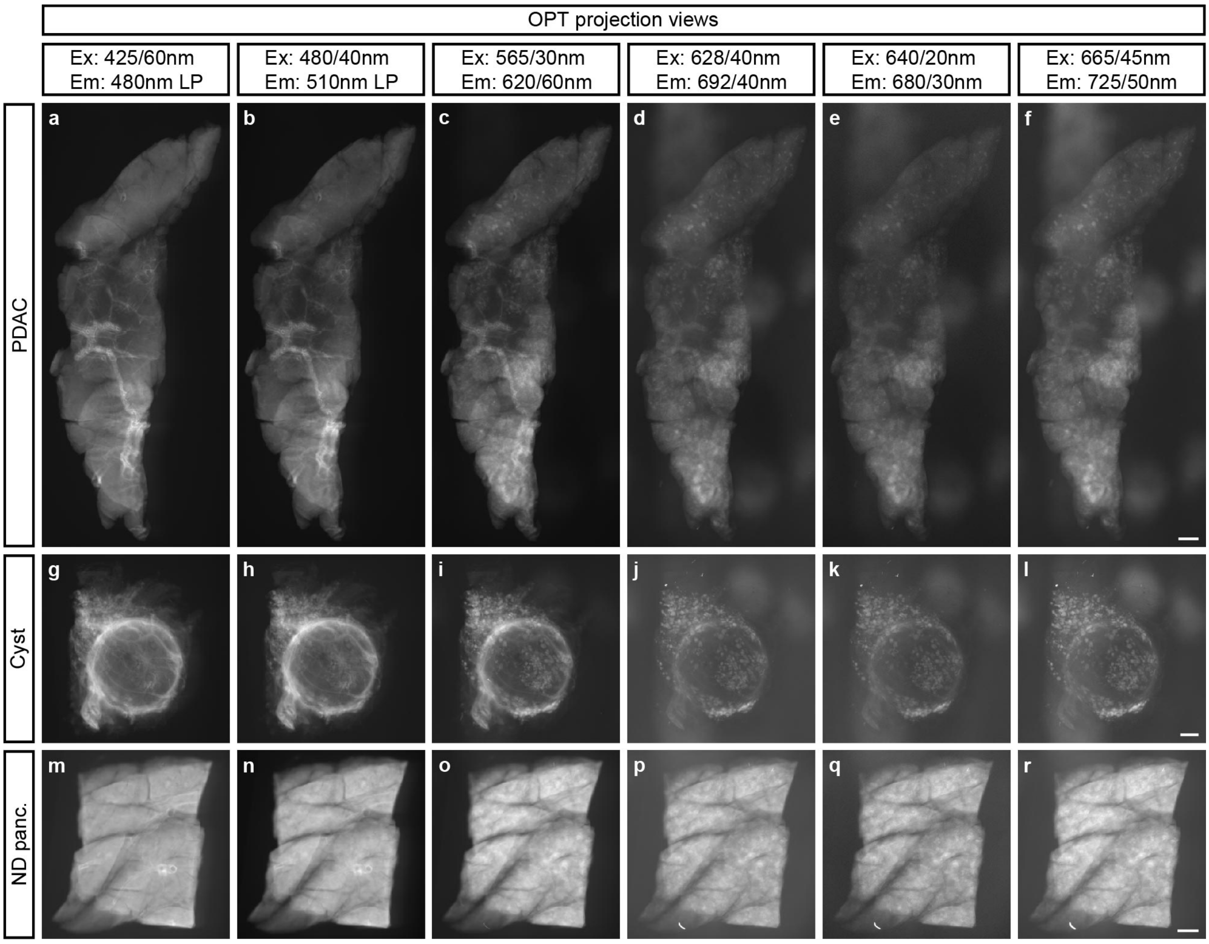

Supplementary Figure 1. Comparative assessment of AF structures captured in different parts of the spectrum. a-f, OPT projection views of the PDAC sample displayed in Fig. 1, captured using indicated filters. **g-l**, OPT projection views of the pancreatic cyst displayed in Fig. 2, captured using indicated filters. **m-r**, OPT projection views of the pancreatic biopsy displayed in Fig. S5, captured using indicated filters. Note, exposure times were the same for all filter sets for all samples (listed in Supplementary Dataset 1). Abbreviations; Ex, Excitation: Em, Emission: LP, Long Pass: ND Panc, Non-diabetic pancreas. Scale bar in (f), (l) and (r) is μm (a-f), (g-l) and (m-r) respectively.

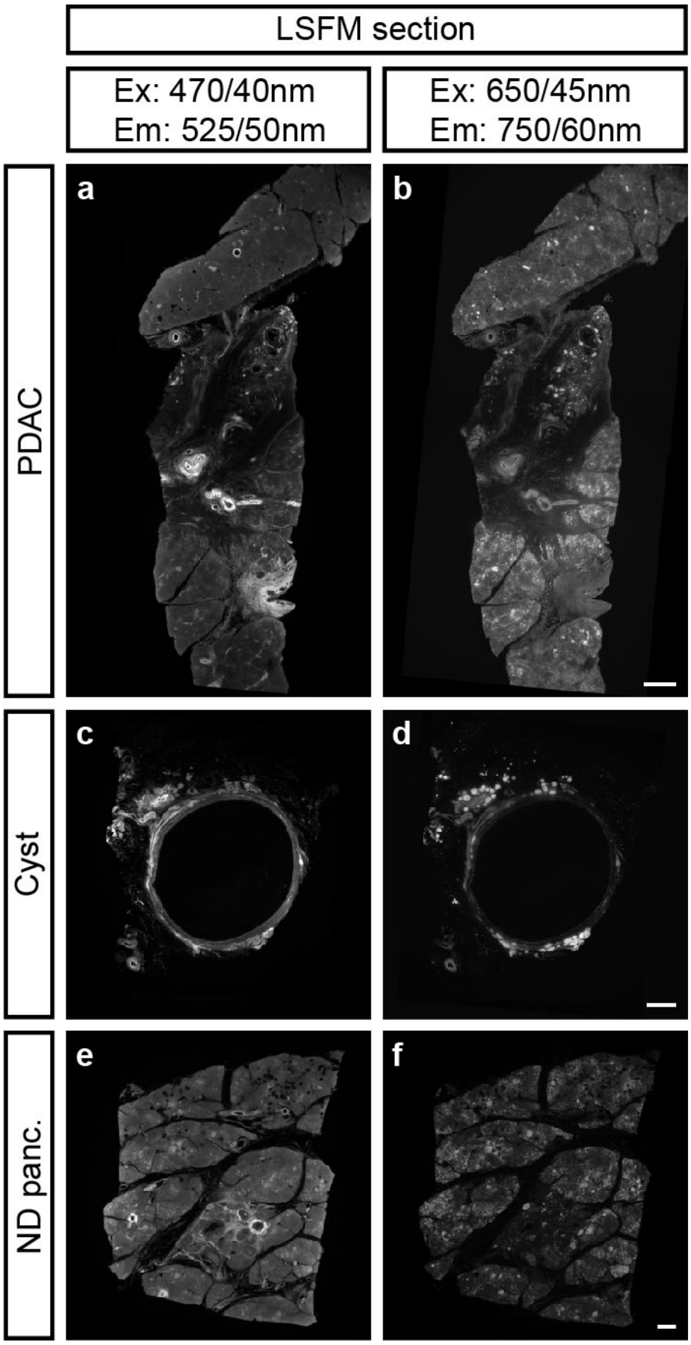

Supplementary Figure 2. **Representative sections of tissue AF from LSFM scans.**

a-f, LSFM images of the samples displayed in Fig. 1 (a, b), Fig. 2 (c, d) and Fig. S5 (e, f), using indicated filter sets. The described tissue processing protocols enable dual modality imaging by OPT and LSFM, allowing to take advantage of the respective imaging modalities strong points. Abbreviations; Ex, Excitation: Em, Emission: ND panc, Non-diabetic pancreas Scale bar in (b, d) is 1000 μm in (a-d), scale bar in (f) is 500 μm in (e, f).

Supplementary movie captions

Supplementary Movie 1. LSFM maximum intensity projection (MIP) movie of the PDAC sample displayed in Fig. 1. **Left**, 3D rotation display with the respective section planes illustrated. **Top right,** z-sections through the y-x plane. **Bottom right,** y-sections through the z-x plane.

Supplementary Movie 2. OPT maximum intensity projection (MIP) movie of the PDAC sample displayed in Fig. 1. **Left**, 3D rotation display with the respective section planes illustrated. **Top right,** z-sections through the y-x plane. **Bottom right,** y-sections through the z-x plane.

Supplementary Movie 3. Rotation and close-up of LSFM maximum intensity projection (MIP) movie of the PDAC sample displayed in Fig. 1.

Supplementary Movie 4. NIR-OPT generated movies of the PDAC tissue seen in Figure 1, displaying left to right; Maximum intensity projection (MIP) view using indicated filter sets (left), iso-surfaced "transparent anatomy" segmentation based on tissue AF in which the delineation of the tumour tissue (grey) is based on low AF intensity (center), and a spot intensity calculation illustrating the range of intensities throughout the sample (right).

Supplementary Movie 5. AF based LSFM maximum intensity projection (MIP) movie of the low grade PanIN displayed in Fig. S4. **Left**, Rotation of the sample with the respective section planes illustrated. **Top right**, y-section going through the z-x plane. **Bottom right**, x-section going through y-z plane.

Supplementary Movie 6. Rotation and close-up of LSFM maximum Intensity projection (MIP) movie of the IPMN cyst displayed in Fig. 2.

Supplementary Movie 7. AF based LSFM maximum intensity projection (MIP) movie of the IPMN cyst displayed in Figure 2. **Left**, 3D rotation display with the respective section planes illustrated. **Top right,** z-sections through the y-x plane. **Bottom right,** y-sections through the z-x plane.

Supplementary Movie 8. AF based OPT maximum intensity projection (MIP) movie of normal pancreatic tissue in different wavelengths, showing tubular structures (left) and islets of Langerhans (right).

Legend Supplementary Dataset 1

Donor information, OPT image acquisition parameters and Imaris settings for image analysis and display.
