# Supplementary figures and images for: Mesoscopic 3D imaging of pancreatic cancer and Langerhans islets based on tissue autofluorescence
Source: Sci Rep. 2020 Oct 26;10:18246. doi: 10.1038/s41598-020-74616-6 (PMC7588461; doi:10.1038/s41598-020-74616-6)

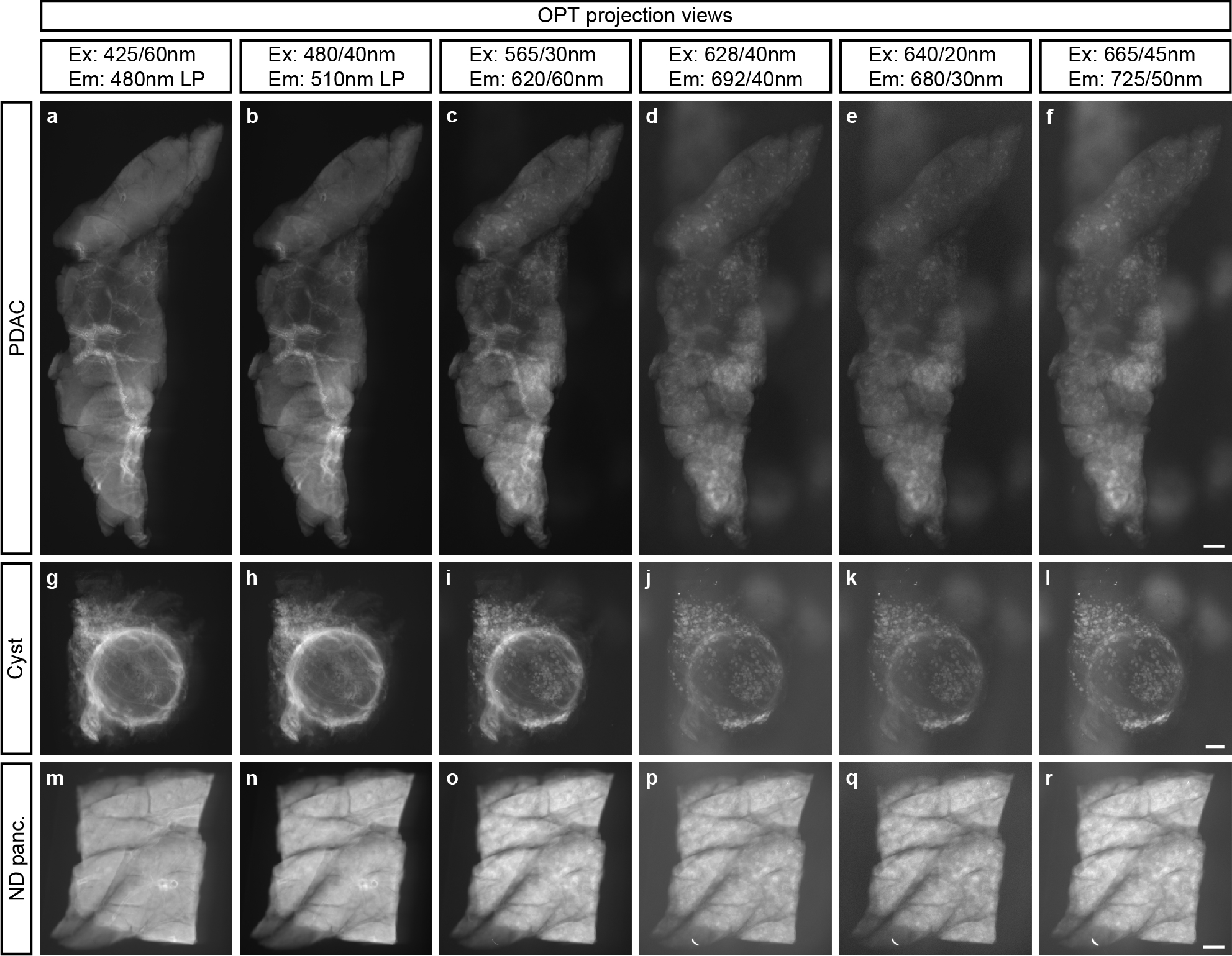

Supplement: Supplementary file 2 — Supplementary Figure S1. [file 41598_2020_74616_MOESM2_ESM.jpg]

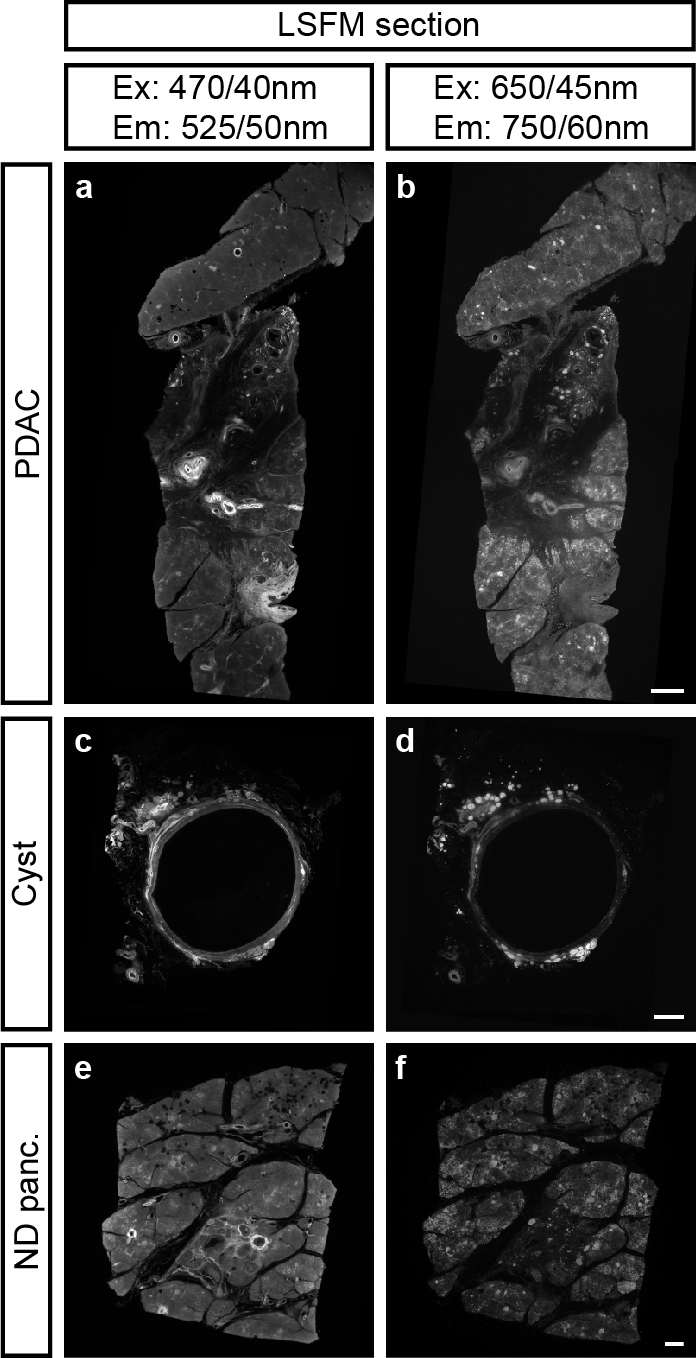

Supplement: Supplementary file 3 — Supplementary Figure S2. [file 41598_2020_74616_MOESM3_ESM.jpg]
